# Polyneuropathy in Gaucher disease type 1 and 3 – a descriptive case series

**DOI:** 10.1038/s41598-019-51976-2

**Published:** 2019-10-25

**Authors:** Mattias Andréasson, Göran Solders, Cecilia K. Björkvall, Maciej Machaczka, Per Svenningsson

**Affiliations:** 1 Department of Neurology, Karolinska University Hospital, Stockholm, Sweden; 2 Center for Neurology, Academic Specialist Center, Stockholm, Sweden; 3 Department of Clinical Neurophysiology, Karolinska University Hospital, Stockholm, Sweden; 4 Department of Medicine, Sunderby Regional Hospital of Norrbotten County, Luleå, Sweden; 5 Medical Faculty, University of Rzeszow, Rzeszow, Poland; 6 Department of Clinical Science and Education, Stockholm South Hospital and Karolinska Institutet, Stockholm, Sweden; 7 Department of Clinical Neuroscience, Karolinska Institutet, Stockholm, Sweden

**Keywords:** Peripheral neuropathies, Neuromuscular disease, Metabolic disorders, Haematological diseases

## Abstract

Polyneuropathy (PNP) has been reported to be a possible phenotypic feature in Gaucher disease type 1 (GD1), while less is known about PNP in type 3 (GD3). We performed a cross-sectional study, exploring PNP in a Swedish GD cohort. Clinical assessment and blood biochemistry were carried out in 8 patients with GD1 and 11 patients with GD3. In patients with symptoms or clinical findings indicative of PNP, nerve conduction studies and quantitative sensory testing were performed. Assessments were compared to historic controls. A subclinical small fiber neuropathy (SFN) was demonstrated in 2 of 8 patients in the significantly (p = 0,021) older GD1 cohort. A large fiber PNP was evident in an additional 3 GD1 patients but could not be ascribed as disease manifestation. No GD3 patients exhibited neurophysiological evidence of small or large fiber PNP attributed to GD3. Compared to historic controls, no significant group differences were evident with regard to neuropathy rating scores. In summary, our study does not support large fiber PNP as a prevalent manifestation of GD. SFN is a possible feature in GD1, although small sample size limits definite conclusions. Our study provides novel data, arguing against clinically significant small or large fiber PNP in GD3.

## Introduction

Gaucher disease (GD) is an autosomal recessive disease, caused by mutations in the *GBA1* gene. This gene encodes the lysosomal enzyme glucocerebrosidase (GCase), which is essential for the metabolism of glycosphingolipids. Enzyme deficiency generates accumulation of its main substrate, glucosylceramide, considered central for disease pathogenesis. GD is thus characterized as a lysosomal storage disorder^1^.

GD is classified into three different forms with regard to phenotypic presentation. GD type 2 (GD2) is characterized by childhood-onset, with aggressive involvement of the central nervous system (CNS), often resulting in death within 2 years. GD type 3 (GD3) presents with a variable disease severity but a mandatory severe CNS involvement^1–3^. GD type 1 (GD1) has historically been viewed as a non-neuropathic phenotype, mainly presenting with symptoms secondary to visceral involvement^2^. However, it is now well-established that both homozygous and heterozygous *GBA1* mutations also confer an increased risk of developing Parkinson’s disease (PD)^4^.

In recent years, retrospective, cross-sectional and prospective studies evaluating involvement of the peripheral nervous system (PNS) in GD1 have been conducted, showing an increased prevalence and incidence of polyneuropathy (PNP)^5–7^. PNP associated with GD1 has been described as axonal, engaging both motor and sensory nerve fibres^7^.

Cobalamin deficiency is a known cause of PNP^8^. The intracellular cobalamin pathways are dependent on the initial cellular uptake of cobalamin into the lysosome, from where further cellular processing ensues^9–11^. An increased susceptibility to functional cobalamin deficiency in GD, precipitated by disease associated lysosomal dysfunction, has been suggested^12^.

We conducted a cross-sectional study, exploring PNP and cobalamin status in a Swedish GD cohort.

## Methods

### Subjects

From November 2017 until August 2018, all adult patients attending regular clinical visits to the Dept. of Hematology, Karolinska University Hospital and the Dept. of Medicine, Sunderby Regional Hospital of Norrbotten County, were invited to participate in the study. All patients had a previously confirmed molecular and genetic diagnosis of GD1 or GD3.

All patients have signed an informed consent and the study was approved by the regional ethical board of Stockholm, Sweden (ref. nr 2016/19-31/1 and 2017/1957-32/1, Regionala Etikprövningsnämnden Stockholm). Patient related investigations were undertaken in accordance with the Helsinki Declaration.

### Clinical assessment

Patients were evaluated by oral interview assessing current medication, disease history, smoking habits and a review of medical records. The presence of heavy alcohol consumption, as outlined by the Swedish National Board of Health and Welfare, was determined by oral history^13^. Standardized questions, with regard to symmetric symptoms compatible with a possible PNP, were undertaken including; “*cold*, *pinching or burning pain-like sensations in extremities*”, “*electric shock-like pain or sensations in extremities*”, “*subjective muscle weakness*” and “*subjective impairment of sensation*”. Clinical examination encompassed the Utah Early Neuropathy Scale (UENS) with the aim of detecting an early sensory predominant PNP^14^. GD3 patients were further evaluated with the modified Severity Scoring Tool (mSST)^15^.

### Biochemistry

Non-fasting venous blood samples were collected for complete blood count, blood differential, creatinine, electrolytes, liver function test, s-electrophoresis, p/s-glucose and/or B-hemoglobin A1c (HbA1c). As part of the targeted assessment of cobalamin status, levels of p/s-homocysteine (Hcy), s-folic acid (folate), s-vitamin B12 (B12) and s-methylmalonic acid (MMA) were determined. Analyses were carried out according to established GMP approved clinical routines at Karolinska University Hospital, Sunderby County Hospital and University Hospital of Umeå.

### Neurophysiology

Patients describing symptoms compatible with PNP, or an UENS score ≥4, were scheduled for electrodiagnostic testing. UENS is considered sensitive in detecting small fiber neuropathy (SFN) and a cut-off value of >4 has been used in previous studies evaluating sensory predominant PNP in patients with PD^14,16,17^. Considering previous phenotypic descriptions of PNP in GD1, including SFN and axonal large fiber sensorimotor PNP, the cut-off value of ≥4 was chosen^7,18^. Testing was performed at three different sites (Stockholm, Sunderby and Umeå) and the protocol consisted of unilateral motor and sensory nerve conduction studies from the median, peroneal, tibial, ulnar and sural nerves. The parameters were carried out with Natus, Viking EDX (Cephalon A/S; Denmark) and compared to normal values corrected for age and/or height. Quantitative sensory testing (QST) was performed bilaterally in feet and unilaterally in hand. At the University Hospital of Umeå, QST was performed with Modular Sensory Analyzer Thermotest (Somedic; Sweden), at Karolinska University Hospital with Medusa, TSA II (Cephalon A/S; Denmark) and at Sunderby Regional Hospital with RollTemp (Somedic; Sweden).

### Controls

Historic controls were used for comparison of UENS scores and biochemical measurements including MMA, B12, Hcy and folate. The controls are part of a previously published study which recruited control subjects aged 55–70 years with no previously known diagnosis of PNP, diabetes mellitus or alcohol overconsumption^17^. In order to generate a sample size comparable to the GD cohorts in the present study, the 11 youngest historic controls were used.

### Statistical analyses

Categorical variables were compared with Fisher’s Exact Test. Non-parametric Mann-Whitney U-test was used for comparison of numerical variables. A two-tailed p-value of <0,05 was considered significant. All statistical analyses were carried out with IBM SPSS Statistics for Windows, version 25.0 (IBM Corp., Armonk, N.Y., USA).

## Results

### GD1 cohort

A summary of the characteristics of the GD1 cohort is shown in Table 1. One patient declined participation and the cohort (n = 8) had a median age of 54 years. Three of the patients (Pt 1, Pt 5, Pt 6) reported symptoms compatible with PNP, along with an UENS score ≥4. An additional two patients (Pt 2, Pt 8) had an UENS score ≥4 without reporting symptoms.

**Table 1:** Clinical, neurophysiological and biochemical characteristics of the GD1 cohort.

Pt.	Age/sex (y/M,F)	Age at Dx (y)	*GBA1* genotype	Therapy	Splen.	DM	Smoker	Alcohol^b^	B12 subst.	Symptoms	UENS (p)	NCS	QST	p-Hcy (µmol/l) [5,0–15]	s-MMA (µmol/l) [ < 0,37]	s-folate (nmol/l) [ > 7]	s-B12 (pmol/l) [150–650]
1^a^	52/F	48	c.604 C > T/c.1226 A > G	ERT	No	No	No	No	Yes	Yes	6	—	—	17*	0.17	11	300
2	61/M	55	c.1226 A > G/c.1226 A > G	ERT	No	No	No	No	No	No	4	Normal	SFN (C-fibers)	18*	0.35	28	150
3	52/M	3	c.437 C > T/c.1226 A > G	ERT	Yes	No	No	No	No	No	0	—	—	13	0.14	12	300
4	29/F	3	c.798 C > G/c.1040 T > G	ERT	Yes	No	Yes	No	Yes	No	0	—	—	6.8	0.06	25	330
5	73/M	60	c.721 G > A/c.1226 A > G	ERT	No	No	No	Yes	No	Yes	11	Mild demyelinating motor PNP	No SFN	23*	0.16	6*	310
6	64/F	54	c.1226 A > G/c.1226 A > G	SRT(miglustat)	No	No	No	No	Yes	Yes	4	Mild demyelinating motor PNP	No SFN	12	0.14	15	>1100
7	48/M	34	c.1226 A > G/c.1448 T > C	ERT	No	No	No	No	No	No	3	Normal^c^	No SFN^c^	11	0.18	15	310
8	55/M	14	c.1226 A > G/c.1226 A > G	None	No	No	No	No	Yes	No	5	Normal	SFN(C-fibers)	7.6	0.12	37	470

Clinical and neurophysiological evidence of a subclinical SFN in Pt 2 and Pt 8 and a symptomatic large fiber PNP in Pt 1, Pt 5 and Pt 6.

Abbreviations: Pt. – patient; y – years; M, F – male, female; Dx – diagnosis; Splen. – splenectomy; DM – diabetes mellitus; B12 subst. – vitamin B12 substitution; Symptoms – subjective symptoms of polyneuropathy; UENS – Utah Early Neuropathy Scale; NCS – nerve conduction studies; QST – quantitative sensory testing; ERT – enzyme replacement therapy; SRT – substrate reduction therapy; Yes/No – indicates the presence (yes) or absence (no) of a specific parameter; PNP – polyneuropathy; SFN – small fiber neuropathy. Footnotes: *Value outside reference range, ^a^Established chemotherapy induced PNP, ^b^≥168 (M) or ≥ 108 (F) gram alcohol/week^13^, ^c^Electrodiagnostic testing performed 9,5 months after baseline.

Patient 1 (Pt 1) was not assessed electrodiagnostically, since an established chemotherapy induced peripheral neuropathy due to lung cancer was present. Neurophysiological evidence of a symptomatic mild demyelinating motor PNP was found in Pt 5 and Pt 6. In Pt 5, an alcohol overconsumption was evident and the patient exhibited a mild folic acid deficiency. Pt 6 received treatment with miglustat and further investigation could not identify any other cause of PNP. The two asymptomatic patients with an UENS score ≥4, Pt 2 and Pt 8, both demonstrated findings on QST compatible with SFN (Table 1).

Mild Hcy elevations were seen in three patients, however none of the GD1 patients showed evidence of functional cobalamin deficiency, as reflected by normal MMA levels (Table 1). No patient demonstrated increased levels of HbA1c or visible monoclonal gammopathy.

### GD3 cohort

A summary of the characteristics of the GD3 cohort is shown in Table 2. No patient declined participation and the cohort (n = 11) had a median age of 39 years. With the exception of Pt 19, all were classified as Norrbottnian GD3. Three of the patients (Pt 14, Pt 16, Pt 17) reported symptoms compatible with PNP of which one had an UENS score ≥4. An additional two patients (Pt 9, Pt 19) had an UENS score ≥4 without reporting symptoms.

**Table 2:** Clinical, neurophysiological and biochemical characteristics of the GD3 cohort.

Pt.	Age/sex (y/M,F)	Age at Dx (y)	*GBA1* genotype	Therapy	Splen.	DM	Smoker	Alcohol^a^	B12 subst.	mSST (p)	Symp-toms	UENS (p)	NCS	QST	p-Hcy (µmol/l) [0–15]	s-MMA (µmol/l) [<0,4]	s-folate (nmol/l) [≥7]	s-B12 (pmol/l) [180–660]
9	32/F	2	c.1448 T > C/c.1448 T > C	BMT	Yes^part.^	No	No	No	Yes	7.5	No	5	—	—	11.4	0.06	13	705
10	39/F	7	c.1448 T > C/c.1448 T > C	ERT	Yes^part.^	No	No	No	No	5	No	3	—	—	16.2*	0.2	13	229
11	29/M	3	c.1448 T > C/c.1448 T > C	ERT	No	No	No	No	No	11.5	No	0	—	—	16.4*	1.12*	9.8	606
12	52/M	5	c.1448 T > C/c.1448 T > C	ERT	Yes	No	No	No	No	15	No	0	—	—	12.2	0.17	6.9*	344
13	44/F	2	c.1448 T > C/c.1448 T > C	BMT	Yes	No	No	No	Yes	18	No	1	—	—	—	0.13	—	879
14	30/M	2	c.1448 T > C/c.1448 T > C	ERT	Yes	No	No	No	No	15	Yes	4	Normal	No SFN	12.5	0.1	11	318
15	51/F	2	c.1448 T > C/c.1448 T > C	ERT	Yes	No	No	No	No	5.5	No	1	—	—	11.9	0.1	14	346
16	57/F	3	c.1448 T > C/c.1448 T > C	ERT	Yes	No	No	No	No	12.5	Yes	3	Mild axonal sensorimotor PNP	No SFN	16.8*	0.24	>45	549
17	51/M	1	c.1448 T > C/c.1448 T > C	ERT	Yes	No	No	No	No	13.5	Yes	1	Normal	No SFN	11.3	0.15	39	420
18	24/M	1	c.1138 G > A/c.1448 T > C	ERT	No	No	No	No	No	1.5	No	0	—	—	10	0.14	13	338
19	18/F	3	c.1448 T > C/c.1448 T > C	ERT + SRT (miglustat)	Yes	No	No	No	No	—	No	≥4^b^	—	—	4.2^c^	0.12^c^	26^c^	470^c^

Pt 16 was symptomatic and was the only subject with electrodiagnostic evidence of a large fiber PNP. Pt 14 and 17 reported symptoms but QST was not compatible with SFN. Pt 9 and 19 had an UENS score ≥4 but electrodiagnostic testing was declined.

Abbreviations: Pt. – patient; y – years; M, F – male, female; Dx – diagnosis; Splen. – splenectomy; DM – diabetes mellitus; B12 subst. – vitamin B12 substitution; mSST - modified Severity Scoring Tool; Symptoms – subjective symptoms of polyneuropathy; UENS – Utah Early Neuropathy Scale; NCS – nerve conduction studies; QST – quantitative sensory testing; ERT – enzyme replacement therapy; SRT – substrate reduction therapy; BMT – bone marrow transplant; Yes/No – indicates the presence (yes) or absence (no) of a specific parameter; yes^part.^- partial splenectomy; PNP – polyneuropathy; SFN – small fiber neuropathy. Footnotes: *Value outside reference range, ^a^≥168 (M) or ≥108 (F) gram alcohol/week^13^, ^b^Absent ankle reflexes, further testing limited due to cognitive impairment, ^c^Reference range as shown in Table 1.

Electrodiagnostic testing was performed in the three symptomatic patients, while Pt 9 declined testing and Pt 19 was considered ethically unsuitable due to cognitive impairment. A mild large fiber PNP was only demonstrated in Pt 16, who suffered from concurrent rheumatoid arthritis and polymyalgia rheumatica treated with prednisolone. QST revealed pathology in Pt 17, who suffered from severe kyphosis, however the distribution of findings was asymmetric and not deemed typical for SFN.

Only one patient, without symptoms or clinical findings, demonstrated an elevation of MMA, fitting with a functional cobalamin deficiency (Table 2). No monoclonal gammopathy was detected and none of the patients with neuropathic symptoms had elevated s-glucose.

### Comparison to historic controls

The control group was matched to the two GD cohorts with regard to gender, but significantly older compared to the GD3 cohort (p < 0,05). No significant differences in UENS scores were seen when comparing controls to GD1 (p = 0,19) and GD3 (p = 0,49). None of the two GD groups had significantly higher MMA levels when compared to controls (Table 3).

**Table 3:** Comparison with historic controls.

	GD1 *(n* = *8)*	GD3 *(n* = 11*)*	Controls *(n* = *11*)	p-value
Age (y), median (IQR)	54 (14)	39 (22)	64 (12)	0.078^a^, **0.021**^b^, **<0.05**^c^
Sex				NS^a,b,c^
*male (%)*	5 (0.63)	5 (0,45)	5 (0,45)	
*female (%)*	3 (0.38)	6 (0.55)	6 (0.55)	
UENS (p), median (IQR)	4 (5)	1 (4)	2 (2)	0.19^a^, 0.18^b^, 0.49^c^
s-MMA (µmol/l) [<0,37], median (IQR)	0.15 (0.05)	0.14 (0.1)	0.2 (0.08)	**0.038**^a^, 0.79^b^, 0.067^c^

A significant lower age in the GD3 cohort, compared to both GD1 and controls, is seen (p = 0,021 and p < 0,05). No significant differences in UENS scores between the three groups. Statistical tests performed with two-tailed Mann-Whitney U-test and Fishers Exact Test.

Abbreviations: IQR – interquartile range, UENS – Utah Early Neuropathy Scale, s-MMA – serum methylmalonic acid, NS – not significant Footnotes: ^a^GD1 compared to controls, ^b^GD1 compared to GD3, ^c^GD3 compared to controls.

## Discussion

The present study, comprising 8 GD1 and 11 GD3 patients, demonstrated two cases interpreted as GD1 associated SFN and no clear case of GD1 or GD3 associated large fiber PNP.

In two GD1 patients with large fiber PNP (Pt 1, Pt 5), the underlying etiology was clouded by co-morbidities and the large fiber PNP found in the third GD1 patient (Pt 6) was confounded by exposure to miglustat. Since the initial treatment study from 2000, miglustat has been debated whether or not it could be causative of PNP^19^. The previously referenced study from 2010 challenged such association by demonstrating an increased prevalence and incidence of PNP in GD1 patients not receiving miglustat^7^. In the present study, we considered miglustat a potential confounder and the demonstrated PNP in Pt 6 was not interpreted as a disease manifestation.

Two GD1 patients exhibited clinical signs and findings on QST compatible with SFN with no other identified cause. Thus, we cannot exclude SFN as a possible phenotypic feature in GD1, although the current study lacks a sufficient sample size do draw firm conclusions. We note that the GD1 cohort tended to have a higher median UENS score compared to the older historic controls, although not significant (p = 0,19) and influenced by the other causes of PNP identified in the GD1 group (Table 3). The absence, in our GD1 cohort, of large fiber PNP as a clear disease manifestation differs from the previously referred study^7^. However, in that study, the sample size was larger and no neurophysiological assessment of SFN was carried out. Thus, we cannot exclude the possibility of co-existent SFN in the PNP cases reported there, which would be in agreement with the SFN cases detected in our study and the study by Devigili *et al*.^18^. A longitudinal follow-up of our GD1 cohort could discern whether patients with SFN are at risk of developing large fiber involvement over time.

Three of eleven patients in the GD3 cohort had symptoms compatible with PNP, of which none demonstrated neurophysiological evidence of SFN. Only one patient, Pt 16, showed evidence of mild large fiber PNP, but co-morbidities prevented the establishment of a causal relationship to GD3.

With regard to PNS involvement in GD3, previous studies are scarce. Eleven patients with Norrbottnian GD3 were evaluated in a study from 1980, including motor nerve conduction studies and electromyography, without signs of PNP. The majority of these patients were under the age of 20 and no assessment of small fiber function was carried out^19^. To our knowledge, no other systematic small and large fiber PNP evaluation has been performed in patients with GD3.

In the present study, a significant (p = 0,021) age difference is seen with a higher median age in the GD1 group compared to GD3. More specifically, the two GD1 patients with SFN were aged 55 and 61 at time of evaluation. Thus, SFN as a late-onset feature in GD1 is a possibility, similarly to what is seen with the development of PD in patients carrying *GBA1* mutations^4,20^. A higher prevalence of PNP in GD3 could have been expected, considering the greater CNS involvement seen in this disease subtype compared to GD1. There are possible explanations to our surprising findings in this regard. As mentioned, the median age was lower in the GD3 cohort why the existence of late-onset SFN as a feature in GD3 cannot be excluded. Furthermore, the molecular mechanisms underlying peripheral neuropathy associated with GD are not well understood, why different pathways generating CNS and PNS pathology respectively are possible. In this context, it is worth mentioning the established association between the *GBA1* variant E326K and PD, a variant that surprisingly does not cause GD in homozygous state^21^. Finally, it is possible that unknown factors attributed to the Norrbottnian form of GD3 are not present in the global GD3 population, thus our results are primarily representative of the Norrbottnian GD3 population.

Our study argues against cobalamin deficiency being a prevalent finding in GD, with only one asymptomatic patient exhibiting functional cobalamin deficiency (Tables 2 and 3). Thus, the study does not support an association between PNP and cobalamin deficiency in GD.

The main limitation of the present study is small sample size, which is attributed to the rarity of the disease. In the UK, a GD prevalence of 1 in 200 000 has been estimated^22^. However, since GD3 represents less than 10% of all GD patients, we believe the size of our GD3 cohort is a strength of our study^1^.

Confounding factors exist in the assessment of SFN with UENS and QST. QST is a subjective method and does not differentiate between peripheral and central sensory tracts, why myelopathy is a possible confounder even though we deemed symmetric findings as a requirement for SFN diagnosis. Furthermore, Pt 16 was examined with the less sensitive RollTemp instrument. Since this patient had other concurrent diseases, we do not believe the interpretation of our main findings would have changed if QST had revealed SFN. Moreover, we cannot exclude the presence of a subclinical PNP in Pt 9 and Pt 19, considering their mildly increased UENS scores. In this context, it is notable that Pt 19 was exposed to miglustat. Finally, electromyography was not part of the study protocol why a full assessment of potential lower motor neuron involvement was not obtained. Neuronal cell loss, including the spinal anterior horn, has previously been described in an infant with severe GD2^23^.

In conclusion, the present study did not identify large fiber PNP as a prevalent feature in GD, but we cannot exclude SFN as a possible disease manifestation of GD1 as proposed in a previous study^18^. This concept is clinically relevant when assessing pain in GD1 patients, since the treatment for neuropathic pain differs from skeletal or inflammatory pain^18^. Furthermore, considering the absence of neurophysiological evidence of small or large fiber PNP attributed to GD3 in our cohort, our study questions whether a clinically significant PNP is evident in, at least, the Norrbottnian form of GD3, despite the pronounced level of CNS disease severity associated with this disease subtype.

Future studies, including objective measures of SFN such as corneal confocal microscopy and skin biopsies, in GD1 and GD3 patients and matched controls, could further discern the prevalence, severity and underlying pathophysiology of PNP in GD.

## Data Availability

The datasets generated during and/or analysed during the current study are available from the corresponding author on reasonable request.
